# Botulinum Toxin Deaths: What is the Fact?

**DOI:** 10.4103/0974-2077.44169

**Published:** 2008

**Authors:** Omprakash HM, Rajendran SC

**Affiliations:** *Consultant Dermatologist, National Skin and Hair Care Clinic, Bangalore, Karnataka, India*

“All things are poisons and there is nothing that is harmless, the dose alone decides that something is no poison” -Paracelsus (1493–1541).

Botulinum toxin type A/B chemodenervation is the most common cosmetic procedure performed world wide with an estimate of nearly 3 million injections per year. But in January 2008, botulinum toxin serotypes- A and B received bad press reviews. This followed a petition by Dr. Sidney Wolfe, Director of Public Citizen, a consumer advocacy organization in the USA, to the US Federal Drug Administration (FDA). The petition stated that there were nearly 180 adverse effects related to botulinum toxin and that nearly 16 reported deaths during 1997–2006.[1] His contention was that “nobody should die from injected Botulinum toxin”. Dr. Sidney sought appropriate action from the FDA and also favoured the issuing of a Black box warning on botulinum toxin injection cartons as well as product information leaflets, highlighting the adverse events of the drug and also sending warning letters about the same to the doctors.

And, not surprisingly, Allergan Inc., manufacturers of botulinum toxin type - A (*Botox*, *Botox Cosmetic*), and Solstice Neurosciences Inc., manufacturers of botulinum toxin type B (*Myobloc*), have put up a strong defence that there has been no death related to cosmetic usage till date.[2]

So, where does the truth lie and what do the facts say?

A literature review of peer-reviewed articles from 1988 till date was performed. We found two classes of adverse events reported so far – (a) transient and benign events and (b) potentially serious events. The transient and benign events included hematoma, injection site pain, intractable headache, ptosis, diplopia and hyperactivity of the local antagonist muscle.[3] The potentially serious events were sequelae due to systemic spread of toxin leading to botulism-like features, starting as dry and red eye, accommodation difficulty, dry mouth, gastrointestinal disturbances, dysphagia, hoarseness and lastly breathing difficulties.[4]

The next question we probed was which were the indications that lead to potentially serious life-threatening symptoms? The review showed that death after Botox administration for cosmetic indications had never been documented with standard approved formulations. Botulinum toxin administration for indications such as dystonia,[5] hyperhydrosis[6] and blepharospasm[7] had been associated with botulism-like symptoms. And, even in these indications, such side effects had happened possibly because the underlying neuromuscular condition myasthenia gravis was not recognized by the injectors. More importantly, except for hyperhydrosis no other dermatological/cosmetic indication had led to these symptoms.

Presently, Botox Cosmetic is the only toxin that is approved for a cosmetic use – the temporary improvement of glabellar lines. According to FDA, Myobloc is approved for cervical dystonia. Botox is also approved for cervical dystonia as well as for strabismus, blepharospasm and primary axillary hyperhidrosis. Further, botulinum toxin is not approved for children below 12 years for any indication.

However, botulinum toxin has been used for many off-label indications.[1] It has been used by neurologists for cerebral palsy[8] though it is not approved for use in children, as mentioned above. With continued knowledge and experience about the drug, the drug continues to be used for newer indications.

Should dermatologists worry about such incidents? What does the FDA state on this subject officially? As to the issue of death, this was mainly attributed to a high dose of more than 6 U of botulinum toxin/kg used in children and other medical indications, which would not apply to cosmetic use.[9] The most commonly reported use of botulinum toxin among these cases was treatment of limb muscle spasticity associated with cerebral palsy. For Botox, doses ranged from 6.25 to 32 U/kg in these cases. For Myobloc, reported doses were from 388 to 625 U/kg. These doses are far higher than those recommended for dermatologic and cosmetic use and hence, when used properly within recommended doses, the drug is safe.

With regards to other adverse events, the FDA is still analyzing the data collected and the final verdict is still awaited.[9] Pending such final recommendations, FDA advises that physicians should be vigilant for post-injection symptoms such as dysphagia, ptosis and shortness of breath. If patients experience any such symptoms, they should seek immediate attention.

Whatever be the ultimate labelling and recommendations for botulinum toxin types- A and B, in the Indian scenario, with many manufacturers from different countries seeking to introduce their products, which may vary with respect to potency, dosage, antigenicity, etc., the dermatologist should be on guard. In particular, the following parameters need to be studied in detail before administration of the drug:[9]

- Product: the potency and antigenicity vary even within a given serotype of different companies. Potency determinations expressed in “Units” or “U” are different among the botulinum toxin products; clinical doses expressed in units are not comparable from one botulinum product to the next.
- Dilution: lesser the dilution, lesser the volume of injection and better safety.
- Dosage: dosage can vary from 1 to 10 U/injection site with regards to Botox but varies with other brands.
- Drug interactions: amino glycoside, chloroquine, aspirin.
- Diffusion: the toxin diffusion is around 3 cm in diameter.
- Dynamics of the muscle: always know the agonist and antagonist for the given area of injection.
- Drug indication: physicians should be particularly careful while administering the drug for hyperhidrosis as this is one dermatological indication for which high doses of the drug are administered and in which serious adverse effects have been reported.
- Be alert to the potential for systemic effects following administration of botulinum toxins, such as dysphagia, dysphonia, weakness, dyspnea or respiratory distress.
- Understand that these effects have been reported as early as 1 day and as late as several weeks after treatment.
